# Challenges in the transition of care for rare connective tissue diseases: results from the 2023 ERN ReCONNET Transition of Care Task Force survey

**DOI:** 10.1093/rap/rkae149

**Published:** 2025-01-11

**Authors:** Edoardo Marrani, Mojca Zajc Avramovic, Diana Marinello, Rosaria Talarico, Chiara Baldini, Eva Collado-González, João Eurico Fonseca, Linda Schraven, Filipa Oliveira Ramos, Paola Triggianese, Arjan Vissink, Marta Mosca, Tadej Avcin, Gabriele Simonini

**Affiliations:** Rheumatology Unit, ERN ReCONNET Center, IRCCS Meyer Children’s Hospital, Firenze, Italy; Department of Allergology, Rheumatology and Clinical Immunology, Children’s Hospital, University Medical Centre Ljubljana, Ljubljana, Slovenia; Rheumatology Unit, ERN ReCONNET Center, Azienda Ospedaliero Universitaria Pisana, Pisa, Italy; Rheumatology Unit, ERN ReCONNET Center, Azienda Ospedaliero Universitaria Pisana, Pisa, Italy; Rheumatology Unit, ERN ReCONNET Center, Azienda Ospedaliero Universitaria Pisana, Pisa, Italy; Asociación Nacional del Síndrome de Ehlers-Danlos e Hiperlaxitud, Murcia, Spain; ERN ReCONNET European Patient Advocacy Group, Azienda Ospedaliero Universitaria Pisana, Azienda Ospedaliero Universitaria Pisana, Pisa, Italy; Instituto de Medicina Molecular, Faculdade de Medicina, Universidade de Lisboa, ULS Santa Maria, Lisbon, Portugal; Federation of European Scleroderma Associations, Saint Maur, Belgium; Unidade de Reumatologia Pediátrica, Hospital Universitário Santa Maria, ULS Santa Maria, Faculdade de Medicina, Universidade de Lisboa, Lisbon, Portugal; Department of Systems Medicine, University of Rome Tor Vergata, Rome, Italy; Department of Oral and Maxillofacial Surgery, University of Groningen and University Medical Center Groningen, Groningen, The Netherlands; Rheumatology Unit, ERN ReCONNET Center, Azienda Ospedaliero Universitaria Pisana, Pisa, Italy; Department of Allergology, Rheumatology and Clinical Immunology, Children’s Hospital, University Medical Centre Ljubljana, Ljubljana, Slovenia; Rheumatology Unit, ERN ReCONNET Center, IRCCS Meyer Children’s Hospital, Firenze, Italy

**Keywords:** transitional care, connective tissue disease, transition of care, paediatric rheumatology

## Abstract

**Objectives:**

Two different European Reference Networks cover CTDs with paediatric onset, the European Reference Network on Rare and Complex Connective Tissue Diseases (ERN ReCONNET) and the European Reference Network on Rare Immunological Disorders (ERN RITA). The transition of care is a significant focus, with ReCONNET centres actively addressing this through updated programs. Despite these efforts, challenges persist. We aimed to inventory transitional care programs for rare CTDs across Europe.

**Methods:**

In April 2023, the ERN ReCONNET Transition of Care Task Force, consisting of expert clinicians, patient advocates and coordination team members, created a survey to assess transitional care practices. The survey was distributed to ERN ReCONNET and ERN RITA centres and responses received by 15 March 2024 were analysed.

**Results:**

A total of 67 responses from 59 centres across 20 European countries were collected. Paediatric rheumatologists typically initiated the transition process (49% of centres). Twenty centres had joint clinics. Despite positive self-assessments of transitional programs, significant limitations were noted. Transition policies varied, with only 40% of centres having a formal standardized policy and less than half of the centres adhering to available transition of care guidelines. Transfer readiness was evaluated using validated questionnaires in 13% of centres, while 29% transitioned patients based solely on age without any readiness assessments. The main challenges included finding adult-oriented centres and the lack of guidelines or engagement from adult centres. Adult healthcare providers also noted a lack of training in adolescent medicine.

**Conclusion:**

The survey highlighted diverse transition practices and resources across centres, with challenges in readiness evaluation and the use of guidelines. Despite these obstacles, respondents rated ongoing transition processes positively. Enhancing patient perspectives in the transition process is crucial to meet their needs during this critical phase.

Key messages

- Standardized transition policies and improved training for adult healthcare providers around adolescent medicine are needed.
- Half of the centres lack established guidelines for the transition process, while nearly one-third do not utilize any tools for assessing readiness for transfer.
- Barriers to a smooth transition include a lack of training in adolescent medicine for adult rheumatology trainees and the time-consuming nature of preparing documentation for the transition.

## Introduction

In the European Union (EU), a rare disease is defined as one affecting <1 person in 2000 inhabitants, and there are >6000 distinct rare diseases impacting up to 36 million EU citizens. To address the challenges posed by these conditions, the European Reference Networks (ERNs) have been established as virtual networks comprising healthcare providers (HCPs). Their primary objective is to facilitate awareness of complex and rare diseases that require highly specialized treatments and care [1].

The ERNs play active roles in reviewing patients’ diagnoses and treatment plans, establish registries, develop clinical guidelines and enhance the exchange of knowledge and expertise among health professionals and patient organizations. Moreover, a process of continuous monitoring is in place to guarantee the presence and maintenance of the highest standards of care.

At this time, two different networks cover CTDs with paediatric onset. The European Reference Network on Rare and Complex Connective Tissue and Musculoskeletal Diseases (ERN ReCONNET) is dedicated to improving patient care in rare CTDs (rCTDs) across Europe [2]. The network addresses 10 rCTDs, including SSc, MCTDs, idiopathic inflammatory myopathies, APS, UCTDs, IgG4-related diseases, relapsing polychondritis, SLE, SS and Ehlers–Danlos syndrome (EDS).

Currently, ERN ReCONNET involves 64 healthcare providers—55 full members and 9 affiliated partners—from >23 European countries (with a list available on the ERN ReCONNET website).

The European Reference Network on Rare Immunological Disorders (ERN RITA), which focuses on immunodeficiency, autoinflammatory and autoimmune diseases, also has a subtheme regarding paediatric rheumatology, thus involving it in the care of paediatric patients with rCTDs. Currently 36 ERN RITA centres specialize in paediatric rheumatology (with a list available on the ERN RITA website).

Despite the lack of high-quality epidemiological data for most of these rare diseases, a significant percentage of the patients experienced disease onset during childhood and adolescence. Complex CTDs with paediatric onset are often life-long diseases and therefore these patients require access to health facilities from childhood through adulthood.

As adolescence and young adulthood is a critical development phase in life, young people need specialized care over this period. Transitional care, which encompasses the planned and synchronized transition of healthcare from paediatric to adult-oriented systems, is crucial for adolescents and young adults [3]. Indeed, the moving of a paediatric patient with a chronic condition to an adult-oriented facility is sometimes associated with a deterioration of the individual’s health status and quality of life [4–6]. Therefore, as the transitional process is a key issue in the management of paediatric-onset chronic disease, specific guidelines and general recommendations have been developed in recent years [7, 8].

In 2017, a joint initiative by the European Alliance of Associations for Rheumatology, EULAR and the Paediatric Rheumatology European Society (PReS) led to the development of standards and recommendations for transitional care in young people affected by childhood rheumatic chronic diseases. These recommendations include 12 statements that any HCP should address when setting up a transition program [9]. However, these statements provide only a holistic framework for transitional care in patients with rCTDs, and their impact on the specific policies of each HCP may be limited.

Despite these recommendations being formulated several years ago, significant limitations still hinder the development of a standardized approach to transitional care in Europe. These recommendations might not be easily applicable due to limited available resources, a lack of training programs in transitional care and cultural differences. Indeed, the proposed recommendations need to be customized to the different national healthcare systems across Europe. Therefore, a direct comparison of the available transition programs might be helpful, as consistent data regarding European transitional care programs for chronic rheumatic diseases are lacking. Furthermore, patient and adult caregiver perspectives on the transition process have not been extensively evaluated and the outcomes of different models of transitional care for rCTDs have only been described in small cohorts [10] or applied to larger cohort in a single healthcare model, such as the National Health Service [11].

In this context, the establishment of ERNs has the potential to foster collaborative efforts in the field of transitional care. The purpose of our survey was to obtain an inventory of the standards of transitional care models adopted among the centres participating in the ERNs dealing with rCTDs.

## Methods

In April 2023, the ERN ReCONNET research working group decided to constitute the Transition of Care Task Force, joining expert clinicians in paediatric (*n* = 5) and adult care (*n* = 5), patient representatives (ePAGs; *n* = 2) and a member of the ERN ReCONNET Coordination Team. The task force developed a 43-item e-survey after three face-to-face meetings. A question was amended if an agreement of >75% had not been reached.

Briefly, the questionnaire was structured in three parts: questions 1–7 enquired about respondent demographics and characteristics of the centre they work in, questions 8–29 and 32 enquired about ongoing transition practice in each centre and questions 30, 31 and 33–43 investigated the opinions of the respondents regarding the optimal transitional care framework.

The completed survey is available as Supplementary Data S1, available at *Rheumatology Advances in Practice* online. The survey was created using the EUSURVEY platform, a web-based platform developed by the European Commission for creating and publishing online surveys. All personal data are stored on the servers of the European Commission’s Data Centre and managed according to General Data Protection Regulation 2016/679. Ethics committee approval was not needed since the survey was completely anonymous and completed by clinicians; patients were not recruited. A consent to data collection for the purpose of clinical research was completed by each respondent at the time of survey response.

In November 2023, to strengthen the results through a comprehensive overview among the ERNs, an official collaboration regarding the dissemination of the survey was established between ERN ReCONNET and ERN RITA. In November 2023, the survey was sent to ERN ReCONNET and ERN RITA centres. The survey was available to be completed online on 27 December 2023 and responses provided through 15 March 2024 were analysed in the current study. In order to avoid duplication bias during the analysis, we limited the responses to questions 1–29 and 32 to one per centre, regardless of the number of respondents from the same centre. When multiple responses from the same centre were available, we prioritized the replies by selecting those from paediatric-oriented physicians, as their input is considered the most valuable contribution regarding the transition process. Additionally, we chose the most complete responses and manually compared the responses from the same centre to maintain data integrity and reduce potential bias. For questions 30, 31 and 33–43, exploring opinions on the transition of care, we included all received responses.

Results have been reported using a descriptive analysis. Data have been reported as frequency distributions and percentages, when required, as well as mean, median, mode and standard deviation for each of the questions. For qualitative data, thematic analysis was performed. Results were reported using the automated word cloud functionality provided by EuSurvey.

## Results

### Respondent demographics and characteristics

A total of 67 respondents completed the survey. The respondents included paediatric [*n* = 27 (40%)] and adult rheumatologists [*n* = 23 (34%)]. Two respondents (3%) were practicing both paediatric and adult rheumatology, six (9%) were internal medicine specialists and four (6%) specialized in adolescent medicine. An additional four (6%) were geneticists working in centres specializing in EDS. One (5%) unspecified allied health professional replied to this survey.

### Overview of centre organization and transitional policies

The organizational models were described for 59 different centres covering 20 European countries. We received responses from 23 (39%) ERN ReCONNET centres, 12 (20%) from ERN RITA centres and 24 (40%) from centres participating in both ERN ReCONNET and ERN RITA. Thirteen (22%) centres offered only adult care, while 12 (20%) centres were paediatric-oriented facilities. The remaining 34 (58%) facilities offered both paediatric and adult care. An overview of patient transitions and referral patterns in healthcare centres for rCTDs is illustrated in Supplementary Tables S1 and S2, available at *Rheumatology Advances in Practice* online.

#### Transition policy, staff involved, tools and resources

In 24 (40%) centres, a formal transition procedure was established and routinely implemented in clinical practice. Three (5%) centres developed a formal procedure, but it was not fully integrated into clinical practice, while 16 (27%) centres implemented a standard procedure in practice, despite lacking a formalized process. In contrast, another 16 (27%) centres indicated that there was no defined, standardized or well-structured approach to transitional care.

Moreover, we inquired about the use of guidelines during the transition. Table 1 illustrates the adoption rates of clinical practice guidelines across various HCPs.

**Table 1.** Adoption and awareness of clinical practice guidelines for patient transition.

Guideline type used during transition	Centres, *n* (%)
Adherence to clinical practice guidelines	24 (40)
Local guidelines	2 (3)
National and local guidelines	6 (11)
International guidelines	8 (13)
Combination of international and local/national guidelines	8 (13)
Lack of adherence to clinical practice guidelines	18 (31)
Not aware of any guidelines	17 (29)

A designated staff member with primary responsibility for coordinating the transition process was clearly identified in 56 (95%) centres. When present, this staff member was usually a doctor [*n* = 49 (83%)], while a nurse specialist was reported in only a minority of the centres [*n* = 7 (12%)]. The paediatric rheumatologist was the key figure responsible for starting the transition process in 48 (81%) centres. In three (5%) centres, the transition of care was initiated by an adult rheumatologist, while a one (1.5%) centre had a physician trained in adolescent medicine. In three (5%) centres a specialized nurse oversaw the initiation of the process. In 3 of the 14 centres specializing in the management of EDS the coordination task was assigned to a clinical geneticist.

A joint clinic between adult and paediatric HCPs was available in 20 (34%) centres. Of these, one (1.5%) centre also offered patients a virtual consultation service, while all the other centres hosted in-person consultations at either the adult or paediatric facility. The range of healthcare professionals participating in the transition is illustrated in Table 2. Table 3 provides the list of topics routinely addressed during the transition; 11 (18%) centre adopted a formal checklist to address them.

**Table 2.** Healthcare professionals involved in the transition of care by centre.

Healthcare professionals	Centres, *n* (%)
Paediatric physician	55 (93)
Adult physician	53 (90)
Nurse	25 (42)
Physiotherapist or occupational therapist	7 (12)
Clinical geneticist	4 (7)
Clinical pharmacist	3 (5)
Social worker	2 (3)
Psychologist	2 (3)
Clinical research assistant	1 (1.5)
Transition coordinator	1 (1.5)
Temporomandibular joint specialist	1 (1.5)
Ophthalmologist	1 (1.5)

**Table 3.** Topic routinely addressed with patients and caregivers during transition (data shown only for topic reported by >50% of centres).

Topic	Centres, *n* (%)
Medications and treatment compliance	54 (91)
Understanding of the disease	51 (86)
Patients’ responsibility for their own health	47 (80)
Work and education	42 (71)
Expectations of adult services	41 (69)
Preference for adult centre	35 (59)
Sexuality and contraception	35 (59)
Mental health and well-being	33 (56)
Fertility and parenthood	31 (52)

Table 4 presents the contents of the transition documentation; complete integration of the electronic health record system between paediatric and adult services was available in 35 (59%) centres.

**Table 4.** Contents of the transition documentation.

Contents of the transition documentation	Centres, *n* (%)
Full medical history	47 (80)
Last visit report	46 (78)
Recent medications	43 (73)
Transition letter	40 (68)
Full medication history	39 (66)
Social habits (smoking, alcohol use)	22 (37)
Specific issues (such as. needle phobia)	19 (32)
Sexual health and contraception	12 (20)
Education/work plan	10 (17)
Psychological report	5 (8)

Only a minority of centres [*n* = 9 (13%)] had dedicated time to prepare the transition documentation and only 17 (29%) centres kept a database of transitioned patients. Psychological support was offered by 20 (34%) centres, with only 9 centres offering such support in both the paediatric and adult centres.

### Patient characteristics: ages, readiness and influencing factors

The majority of centres [*n* = 37 (63%)] initiated the transition of care for patients 15–18 years of age. Notably, 16 (27%) centres commenced transition after the age of 18 years, while 7 (12%) centres initiated it between the ages of 13 and 15 years. In three (5%) centres, transition began as early as 10–13 years of age.

As expected, the definitive transfer of a patient to an adult-oriented centre occurred between the ages of 18 and 20 years in most centres [*n* = 37 (63%)]. In 18 (30%) centres, the transition process ends between 16 and 18 years of age, while in only 2 (3%) centres it occurs after 20 years of age and in a single institution (1.5%), it occurs before 16 years of age.

The evaluation of transfer readiness by using validated questionnaires was performed in only 8 (13%) centres, while in 17 (29%) centres the transition process depends only on the age of the patient without any standardized assessment of transfer readiness. In 27 (46%) centres, personal experience of the treating physician was the principal factor in determining readiness to transfer. In contrast, only a single centre (1.5%) adopted a formal assessment performed by a psychologist.

### Exploring personal perspectives: respondents’ views on transitional care

In the last section of the questionnaire (questions 30, 31, 33–43), we asked respondents for their individual opinions about transitional care. Thus we included all the responses received (*n* = 67).

### Perspective of paediatric-oriented HCPs

The respondents taking care of paediatric patients [*n* = 34 (51%)] reported difficulties in finding an adult-oriented HCP to transfer patients in only eight cases (23%). The diseases for which difficulties in identifying a referral structure were mentioned were mainly EDS [*n* = 8 (23%)] and idiopathic inflammatory myopathies [*n* = 4 (12%)].

The main difficulties in finding an adult-oriented HCP to transfer patients was the lack of guidelines [*n* = 15 (44%)] and the lack of engagement from specialized adult centres [*n* = 15 (44%)]. Another factor was the lack of a personal connection between the two teams [*n* = 8 (23%)] and the lack of specialized adult centres [*n* = 7 (20%)].

### Perspective of adult-oriented HCPs

We surveyed the health professionals responsible for adult patients [*n* = 43 (64%)] regarding difficulties during transition. Seventeen respondents (39.5%) reported implementing different follow-up practices based on whether the patient had juvenile- or adult-onset disease. Specifically, 22 (51%) stated experiencing differences in disease taxonomy, disease activity assessment and disease damage assessment. Additionally, 24 (56%) reported differences in therapeutic approaches for these patients.

### HCP opinion about transitional care

All 67 respondents were asked to evaluate the effectiveness of the ongoing transition process at their centre. Using a Likert scale ranging from 0 (not satisfied at all) to 10 (completely satisfied), the median score was 7 (s.d. 1.88), with only 17 (25%) respondents rating it <6. Subsequently we explored the perceived weaknesses of the ongoing transition program. ‘Absence of integrated computer systems making documentation preparation too time-consuming’ emerged as the primary difficulty [*n* = 14 (21%)], followed by ‘Not enough/no designated time to prepare documentation’ [*n* = 14 (15%)], ‘lack of guidelines’ [*n* = 8 (11%)] and ‘Absence of validated tools to assess outcomes for adult patients with paediatric-onset diseases’ [*n* = 8 (11%)].

Regarding the optimal age for starting transitional care, the median age suggested was 14.5 years (s.d. 1.72); six respondents (24%) indicated that transition should commence at or before the age of 14 years. However, when asked about the optimal age for transfer, the suggested median age was 18.25 years (s.d. 1.09), with 19 respondents (28%) proposing to postpone the transfer until an age of ≥19 years.

**Figure 1.**
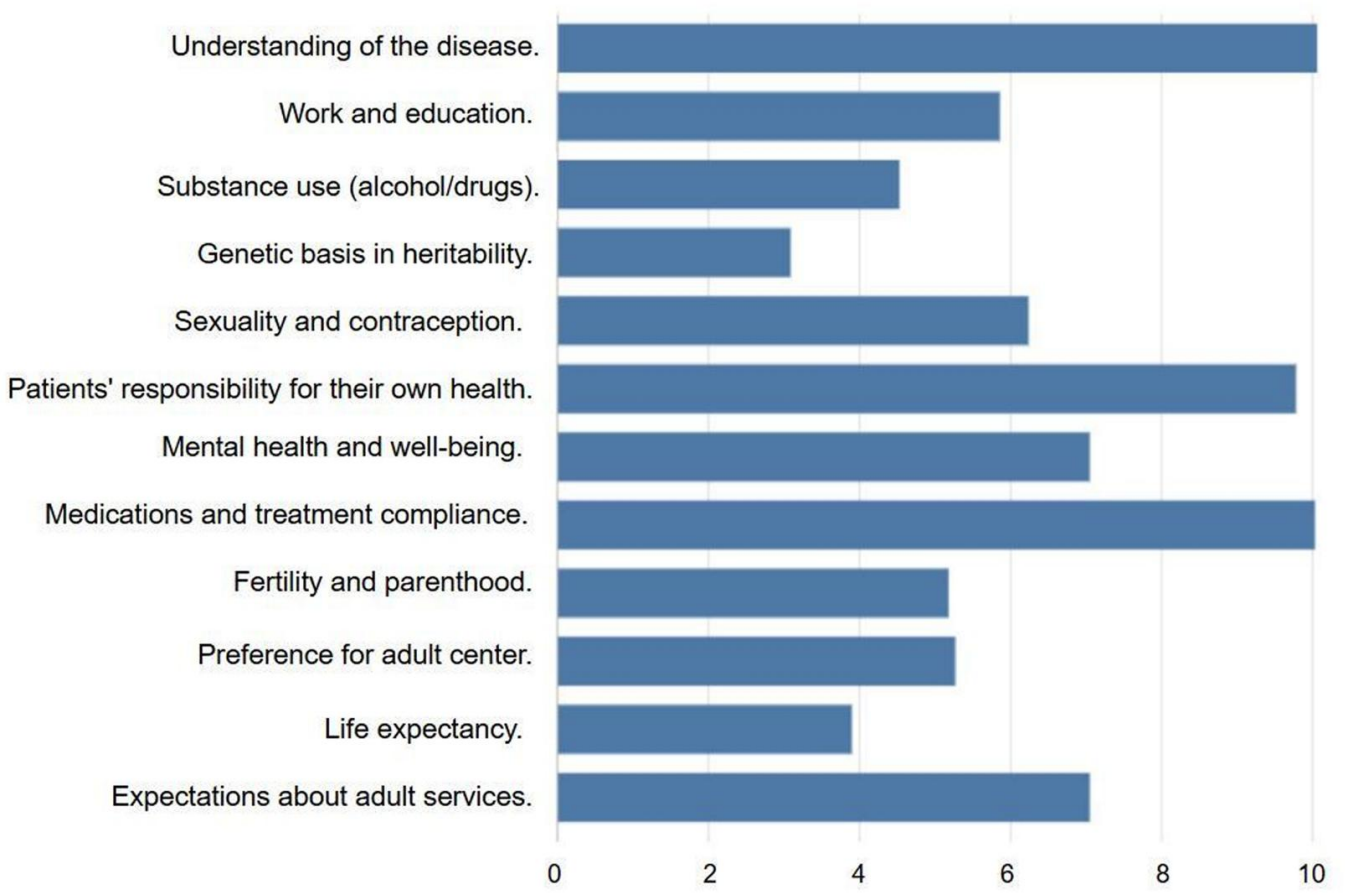
The relevance of the main topic to be discussed with patients and caregivers during transition. Relevance was a score from 0 (least relevant) to 10 (most relevant)

**Figure 2.**
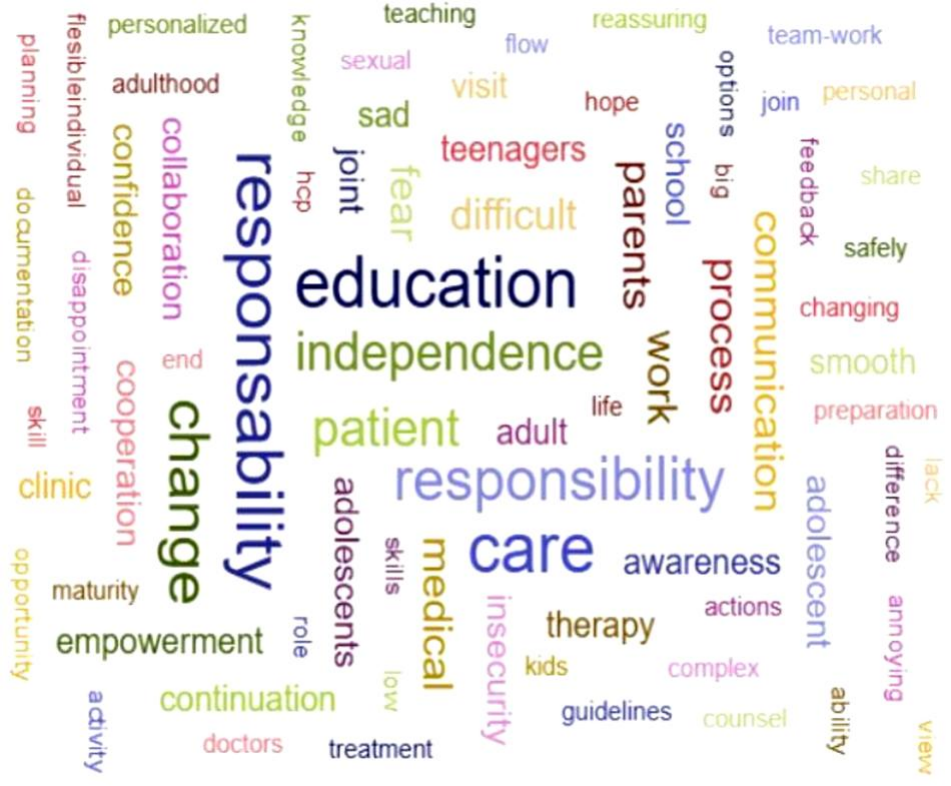
Word cloud showing the first three words that came to mind when thinking about ‘transition’, according to the survey respondents

We also asked about the main topic to be discussed with patients and caregivers during transition (Fig. 1). When asked to provide the first three words that come to mind when thinking of ‘transition’, respondents primarily mentioned ‘responsibility’, ‘education’ and ‘independence’, as illustrated in Fig. 2.

## Discussion

The transition of paediatric patients affected by chronic rheumatic diseases should be regarded as more than a mere administrative task; it should be recognized as an active and coordinated process [7]. A successful transition of care can significantly impact the long-term outcomes of these patients, as a high rate of missed presentations to adult services remains an issue and has detrimental effects on disease activity and related damage [12, 13]. At the same time, the establishment of transitional programs might positively affect the health status of adolescents and young adults with chronic rheumatic disorders [14]. The results of this survey provide a consistent and representative overview of the current landscape regarding transitional programs across Europe.

This survey confirmed the importance of multidisciplinary collaboration in ensuring a successful transition of care. Multidisciplinary collaboration is conducted in a joint clinic, as most centres have already been established, and requires the active participation of various medical and non-medical stakeholders. Compared with more prevalent rheumatic diseases, such as JIA, a crucial aspect of successful transition for complex CTD patients is access, upon transfer, to renal, neurology, respiratory and dermatology services that work alongside rheumatologists. In our survey, respondents referred only to ‘adult’ or ‘paediatric’ physicians as participating in the transition process, without specifying subspecialties. Therefore, in clinical practice, the role of non-rheumatology specialists in the transition process for these patients may not be widespread and needs further elucidation, as cross-specialty collaboration is essential for improving transition outcomes and ensuring continuity of care.

The majority of the respondents rate the ongoing transition process positively, with only one-quarter reporting an insufficient grade. This contrasts with a recent survey from South America, where only 20% of respondents were satisfied with the current transitional program [15].

However, despite an overall positive self-assessment of existing transition programs, the survey highlighted several challenges in implementing effective transitional care. For instance, there is a notable absence of standardized processes in about one-third of the centres, and another one-third lack a formal procedure. The percentage of HCPs adopting a formal, standardized transition policy (40%) is only slightly higher than the results of a previous survey conducted in 2016 across all centres participating in the Paediatric Rheumatology International Trials Organization network, which found that only 27% had a written policy and 46% followed an informal standard procedure [16]. Despite the specific focus of the ERN on the presence and effectiveness of policies related to transitional care and the long interval between the two surveys, significant effort is still needed to guarantee implementation of such procedures.

Moreover, more than half of the centres did not adhere to any established guidelines for the transition process, and a significant minority (29% of centres) did not utilize any tools for assessing readiness for transfer. These findings underscore the urgent necessity for a standardized protocol to ensure seamless transitions for paediatric patients with rare and complex rheumatic diseases.

Theoretically, the EULAR/PReS guidelines should provide a valuable framework for guiding transition care practices, emphasizing the importance of multidisciplinary collaboration, patient-centred approaches and continuous evaluation of outcomes [9]. However, our data indicate that these guidelines are not consistently implemented across centres, and three respondents commented that recommendations were not fully adaptable to local organizations and were too general to be applied to rCTDs.

This issue was also observed in a previous survey conducted by ERN RITA between centres treating immune deficiency and autoinflammatory diseases [17]. Furthermore, issues such as the absence of integrated computer systems, insufficient time for documentation preparation and the lack of validated tools for assessing outcomes are significant barriers to effective transitional care, potentially impacting continuity of care.

Once again, emphasizing the importance of continuous dialogue between adult and paediatric specialists is crucial to establish a common language. Indeed, half of the adult rheumatologists reported differences in disease classification, disease activity assessment and disease damage assessment. Therefore, joint initiatives between adult and paediatric HCPs are necessary to overcome this limitation and develop shared clinical tools and educational resources. Indeed, adult rheumatologists responding to this survey expressed concerns about the lack of training in adolescent medicine and highlighted educational gaps, as also reported in previous surveys [18, 19].

A noteworthy aspect highlighted by the survey is the variability in the age at which transition processes begin and end across different centres. While most respondents suggest starting transition at ≈15 years of age and completing it by 18–20 years, there is considerable variation, with some advocating for earlier or later initiation and completion of the transition process. Once again, this variability underscores the need for standardized guidelines and protocols to ensure consistency and continuity of care across centres. Furthermore, early starting of transition of care has been advocated [20–22]; however, most of the existing research on the benefits of early transition initiation stems from studies on JIA, where disease onset typically occurs at an earlier age. In contrast, for most rCTDs, disease onset often happens later, generally after puberty. This difference suggests that patients with rCTDs may face unique challenges during their transition, as they are diagnosed during a developmental period already marked by significant physical and psychological changes. This may necessitate a more individualized approach to transition timing rather than adhering strictly to early initiation.

The importance of patient empowerment in a successful transition of care process is emphasized by guidelines [8, 9]. Indeed, the survey respondents reported that patients’ responsibility for their own health is a key aspect to be addressed during the transition. ‘Responsibility’ is the primary word chosen by respondents when asked to associate three words with transition of care. However, in practice, it is not always clear how much patients contribute to distinct phases of the process. Transfer readiness is not regularly assessed and patients are often not involved in decisions about choosing an adult-oriented centre or determining the timing of the transfer. Specific patient-reported outcomes regarding the transitional care program for rCTDs represents an urgent unmet need [13, 23], as the few studies were conducted only in cohorts of patients with JIA [20, 24] or in monocentric cohorts of patients with CTDs [25, 26].

Although the high rate of non-presentation to adult services is a critical concern [4–6] and is considered only as an indicator of a lack of patient empowerment, a significant number of respondents, focusing on practical problems faced in daily practice, commented that a structured and well-organized transition program is essential for successful transition to adult facilities. They indicated that non-participation in adult services depends not only on patient engagement, but can also be hindered by organizational problems within healthcare systems.

In conclusion, our survey has offered valuable insights into the current state of transitional care for patients with rare and complex rheumatic and musculoskeletal diseases in Europe. A major limitation of our study is that it was conducted anonymously. As a result, we cannot determine whether the respondents from specific centres are directly involved in the transition process. This anonymity may lead to an underreporting of the available policies for specific HCPs. However, the lack of awareness regarding established policies in a centre highlights the disconnect between formal procedures and routine practices within institutions.

By highlighting strengths, weaknesses and areas for improvement, the task force aims to foster collaboration and knowledge sharing among healthcare professionals to enhance the quality of care and outcomes for transitioning patients. Moreover, as effective transition programs should include robust patient engagement strategies, the next step for the ERN ReCONNET Transition of Care Task Force is to develop a questionnaire for patients and caregivers designed to assess their perspectives, experiences and satisfaction with transition programs. This approach aims to align patients’ and caregivers’ expectations regarding the transition of care, facilitating the development of a shared clinical pathway and a co-designed transition program that can be implemented across our network. Given that patients with rCTDs have unique needs, existing transition frameworks for more prevalent conditions, such as JIA, may not fully address the specific challenges these patients face, despite providing valuable guidance. For this reason, collaborative efforts involving HCPs and patient representatives are critical in designing disease-specific protocols. By addressing both patient empowerment and organizational factors, we can work to minimize missed presentations and improve patient outcomes and retention rates both before and after transition. Ongoing efforts, such as those undertaken by the ERN ReCONNET Transition of Care Task Force, will play a crucial role in shaping these future frameworks, ensuring that they are grounded in the realities of clinical practice and patients’ lived experiences.

## Supplementary Material

rkae149_Supplementary_Data

## Data Availability

The data underlying this article will be shared upon reasonable request to the corresponding author.

## References

[1] Azzopardi-Muscat N , Brand H. Will European Reference Networks herald a new era of care for patients with rare and complex diseases? Eur J Public Health 2015;25:362–3. 25999460 10.1093/eurpub/cku144

[2] Mosca M , Cutolo M. Clinical practice guidelines: the first year of activity of the European Reference Network on Rare and Complex Connective Tissue and Musculoskeletal Diseases (ERN ReCONNET). RMD Open 2018;4:e000791. 30402276 10.1136/rmdopen-2018-000791 PMC6203103

[3] Eleftheriou D , Isenberg DA, Wedderburn LR et al The coming of age of adolescent rheumatology. Nat Rev Rheumatol 2014;10:187–93. 24394351 10.1038/nrrheum.2013.202

[4] Kipps S , Bahu T, Ong K et al Current methods of transfer of young people with type 1 diabetes to adult services. Diabetic Med 2002;19:649–54. 12147145 10.1046/j.1464-5491.2002.00757.x

[5] Yeung E , Kay J, Roosevelt GE et al Lapse of care as a predictor for morbidity in adults with congenital heart disease. Int J Cardiol 2008;125:62–5. 17442438 10.1016/j.ijcard.2007.02.023

[6] Mulla I , Yeung RSM, Noone DG et al Paediatric-to-adult transition experience in vasculitis: report of a model of care and outcomes. Clin Exp Rheumatol 2022;40:772–8. 35238755 10.55563/clinexprheumatol/uhsrnm

[7] Blum RW , Garell D, Hodgman CH et al Transition from child-centered to adult health-care systems for adolescents with chronic conditions. A position paper of the Society for Adolescent Medicine. J Adolesc Health 1993;14:570–6. 8312295 10.1016/1054-139x(93)90143-d

[8] Suris JC , Akre C. Key elements for, and indicators of, a successful transition: an international Delphi study. J Adolesc Health 2015;56:612–8. 26003575 10.1016/j.jadohealth.2015.02.007

[9] Foster HE , Minden K, Clemente D et al EULAR/PReS standards and recommendations for the transitional care of young people with juvenile-onset rheumatic diseases. Ann Rheum Dis 2017;76:639–46. 27802961 10.1136/annrheumdis-2016-210112

[10] García-Rodríguez F , Raygoza-Cortez K, Moreno-Hernandez L et al Outcomes of transitional care programs on adolescent chronic inflammatory systemic diseases: systematic review and meta-analyses. Pediatr Rheumatol 2022;20:15. 10.1186/s12969-022-00670-1 PMC8851760 35177101

[11] McDonagh JE , Hackett J, McGee M, Southwood T, Shaw KL. The evidence base for transition is bigger than you might think. Arch Dis Child Educ Pract Ed 2015;100:321–2. 26310957 10.1136/archdischild-2015-309204

[12] Hazel E , Zhang X, Duffy CM et al High rates of unsuccessful transfer to adult care among young adults with juvenile idiopathic arthritis. Pediatr Rheumatol 2010;8:2. 10.1186/1546-0096-8-2 PMC2820032 20148143

[13] Hersh A , von Scheven E, Yelin E. Adult outcomes of childhood- onset rheumatic diseases. Nat Rev Rheumatol 2011;7:290–5. 21487383 10.1038/nrrheum.2011.38 PMC3705738

[14] Bitencourt N , Lawson E, Bridges J et al Pediatric to adult transition literature: scoping review and rheumatology research prioritization survey results. J Rheumatol 2022;49:1201–13. 35914787 10.3899/jrheum.220262

[15] Borgia RE , De Cunto CL, Terreri MT et al Transition from pediatric to adult rheumatology care: an exploratory study from Latin America. J Clin Rheumatol 2024. doi: 10.1097/RHU.0000000000002055. 38206911

[16] Clemente D , Leon L, Foster H et al Transitional care for rheumatic conditions in Europe: current clinical practice and available resources. Pediatr Rheumatol Online J 2017;15:49. 28599656 10.1186/s12969-017-0179-8 PMC5466791

[17] Israni M , Nicholson B, Mahlaoui N et al Current transition practice for primary immunodeficiencies and autoinflammatory diseases in Europe: a RITA-ERN survey. J Clin Immunol 2023;43:206–16. 36222999 10.1007/s10875-022-01345-y PMC9840587

[18] McDonagh JE , Southwood TR, Shaw KL. Unmet education and training needs of rheumatology health professionals in adolescent health and transitional care. Rheumatology (Oxford) 2004;43:737–43. 14997008 10.1093/rheumatology/keh163

[19] Zisman D , Samad A, Ardoin SP et al US adult rheumatologists’ perspectives on the transition process for young adults with rheumatic conditions. Arthritis Care Res (Hoboken) 2020;72:432–40. 30740937 10.1002/acr.23845

[20] Shaw KL , Southwood TR, McDonagh JE. Young people’s satisfaction of transitional care in adolescent rheumatology in the UK. Child Care Health Dev 2007;33:368–79. 17584391 10.1111/j.1365-2214.2006.00698.x

[21] Nagra A , McGinnity PM, Davis N, Salmon AP. Implementing transition: ready steady go. Arch Dis Child Educ Pract Ed 2015;100:313–20. 26063244 10.1136/archdischild-2014-307423 PMC4680199

[22] Akre C , Suris JC, Belot A et al Building a transitional care checklist in rheumatology: a Delphi-like survey. Joint Bone Spine 2018;85:435–40. 28965941 10.1016/j.jbspin.2017.09.003

[23] Jiang I , Major G, Singh-Grewal D et al Patient and parent perspectives on transition from paediatric to adult healthcare in rheumatic diseases: an interview study. BMJ Open 2021;11:e039670. 10.1136/bmjopen-2020-039670 PMC7783517 33397662

[24] Smitherman EA , Chahine RA, Bitencourt N et al Patient-reported outcomes among transition-age young adults with juvenile idiopathic arthritis in the childhood arthritis and rheumatology research alliance registry. J Rheumatol 2023;50:98–106. 36109074 10.3899/jrheum.220514

[25] Walter M , Hazes JM, Dolhain RJ et al Development of a clinical transition pathway for adolescents in the Netherlands. Nurs Child Young People 2017;29:37–43. 29115764 10.7748/ncyp.2017.e932

[26] Walter M , Kamphuis S, van Pelt P et al Successful implementation of a clinical transition pathway for adolescents with juvenile-onset rheumatic and musculoskeletal diseases. Pediatr Rheumatol 2018;16:50. 10.1186/s12969-018-0268-3 PMC6091100 30075795

