# Supplementary material for: Challenges in the transition of care for rare connective tissue diseases: results from the 2023 ERN ReCONNET Transition of Care Task Force survey
Source: Rheumatol Adv Pract. 2025 Jan 11;9(1):rkae149. doi: 10.1093/rap/rkae149 (PMC11780841; doi:10.1093/rap/rkae149)
Supplement: rkae149_Supplementary_Data [file rkae149_supplementary_data.zip › 24-137 Supplementary Tables.docx]

### **Supplementary Table S1**. Annual Patient Transfers from Pediatric to Adult-Oriented HCPs

| **Number of Patients Transferred Annually** | **Number of Centers** | **(%)** |
| --- | --- | --- |
| >50 patients | 7 | 12 |
| 20-50 patients | 18 | 30 |
| 5-20 patients | 26 | 44 |
| < 5 patients | 7 | 12 |
| Data unavailable | 1 | 1.5 |

**Supplementary Table S2**: Availability of Adult-Oriented HCPs and Referral Patterns for Transition.

| **Category** | **Description** | **Number of Centers** | **(%)** |
| --- | --- | --- | --- |
| **Adult-Oriented HCPs Available for transfer by the pediatric HCPs (n=46)** | Single adult-oriented HCP | 6 | 13 |
|  | 2-5 adult-oriented HCPs | 16 | 35 |
|  | More than 5 adult-oriented HCPs | 4 | 9 |
|  | Data unavailable | 20 | 43 |
| **Specialized Referrals** | adolescent and young adult clinics | 4 | 7 |
|  | clinical geneticists (EDS expertise) | 2 | 3 |
|  | Adult facilities receiving referrals from only 1 center | 17 | 36 |
| **Referral Sources for Adult Facilities (n=47)** | Adult facilities receiving referrals from 2-5 centers | 19 | 40 |
|  | Adult facilities receiving referrals from more than 5 centers | 4 | 9 |
|  | Data unavailable | 7 | 15 |

EDS= *Ehlers*-*Danlos Syndrome*
