# Supplementary material for: Challenges in the transition of care for rare connective tissue diseases: results from the 2023 ERN ReCONNET Transition of Care Task Force survey
Source: Rheumatol Adv Pract. 2025 Jan 11;9(1):rkae149. doi: 10.1093/rap/rkae149 (PMC11780841; doi:10.1093/rap/rkae149)
Supplement: rkae149_Supplementary_Data [file rkae149_supplementary_data.zip › 24-137 Supplementary Data S1. Survey.pdf]

European Reference Network for rare or low prevalence complex diseases
Network Connective Tissue and Musculoskeletal Diseases (ERN ReCONNET)

ERN ReCONNET Transition of care survey for HCPs

Fields marked with * are mandatory.

Disclaimer
The European Commission is not responsible for the content of questionnaires created using the EUSurvey service - it remains the sole responsibility of the form creator and manager. The use of EUSurvey service does not imply a recommendation or endorsement, by the European Commission, of the views expressed within them.

Anonymous mode
The anonymous option has been activated. As a result, your contribution to this survey will be anonymous as the system will not save any personal data such as your IP address.

Introduction

Dear Healthcare Providers,
We cordially invite you to participate in a survey with the goal of enhancing the transitional care experience for individuals living with rare connective tissue diseases (rCTDs). The transition from pediatric-oriented healthcare providers to adult care is a critical phase for individuals affected by rCTDs. As ERN ReCONNET, our primary mission is to ensure the highest standard of care within the healthcare providers (HCPs) that are part of our network. We recognize that existing transitional models may have limitations and may not fully meet our patients' needs. Our aim is to map the transitional models that are currently in use. In parallel, a survey will be distributed to patients to gather feedback on their needs and expectations. The input from both healthcare providers and patients or caregivers will be crucial in the development of a more effective transitional care model to be adopted by ERN ReCONNET's members.
Please note that this survey is anticipated to take approximately 20 minutes to complete. We understand your busy schedules and commitments, and should you need to complete it in multiple sessions, you are welcome to start the survey and return to it later. Your progress will be saved, allowing you to pick up from where you left off at your convenience.

CONSENT STATEMENT
We want to assure you that there are no right or wrong answers, and your responses will not be subject to judgment.
The survey results will be examined in an aggregated and anonymous manner, and the findings will be disseminated to the medical community and the public through presentations at conferences and publications. If you agree to participate, please continue with the survey. If you choose not to participate, you can simply close the survey window or navigate away from this page. Thank you for your willingness to share your valuable perspective with us. Together, we can positively impact the lives of individuals living with rCTDs.

Survey

In responding to this survey, please consider the following definitions:

1. **Transitional care (or transition)** from a pediatric to an adult-oriented HCP refers to a structured and patient-centered active process that prepare adolescents/young adults for the differences between child and adult services, supporting the development of health literacy and self-management skills. This process might start at an earlier age and aims to ensure the continuity of care while addressing the physical, emotional, and psychosocial needs of the patient during this critical period of transition.
2. **Transfer** of a pediatric patient is primarily an administrative event, occurring when the patient reaches an age where they are no longer considered pediatric, typically around 18 years old. At this point, patients are discharged from a pediatric Health Care Provider (HCP) and referred to an adult-oriented HCP.

* Please state the name of your institution:

* Please state your country:

* Which of the following rare or complex connective tissue disease (CTDs) do you treat?
Select all options you consider relevant

Views
Standard Accessibility Mode

Languages
English

Contact
Contact Form (/eusurvey/runner/contactform/ReCONNET-TransitionHCP)

Report abuse (/eusurvey/home/reportAbuse?survey=747496)

- ☐ Antiphospholipid syndrome (APS)
- ☐ Ehlers-Danlos syndromes (EDS)
- ☐ Idiopathic inflammatory myopathies (IIM)
- ☐ IgG4-related diseases (IgG4)
- ☐ Mixed connective tissue diseases (MCTD)
- ☐ Relapsing polychondritis (RP)
- ☐ Sjögren's Disease (SD)
- ☐ Systemic lupus erythematosus (SLE)
- ☐ Systemic Sclerosis (SS)

* Please state if your center is a full member or an affiliated partner of any European Reference Network (ERN) for one or more of rCTDs reported above

- ○ Yes
- ○ No
- ○ I do not know

* Are you:

- ○ adolescent rheumatologist
- ○ adult rheumatologist
- ○ genetician
- ○ internal medicine specialist
- ○ Other healthcare professional (e.g. nurse, physiotherapist, etc)
- ○ pediatric rheumatologist
- ○ rheumatologist taking care of both pediatric and adult patients

* Does your center provide adult or pediatric care?

- ○ Only pediatric care
- ○ Only adult care
- ○ Both pediatric and adult care

* Which patients do you *personally* take care of?

- ○ Only pediatric patients
- ○ Only adult patients
- ○ Both pediatric and adult patients

The first part of the questionnaire refers to the care provided in your center.

*
How many patients are transferred from pediatric to adult-oriented HCPs (including either an adolescent clinic or a adult rheumatology unit) yearly?

- ○ Less than 5
- ○ 5-20
- ○ 20-50
- ○ More than 50
- ○ I do not know

* Do you have a **defined process** for transition? ❓

- ○ Yes
- ○ No
- ○ The process is defined with a formal procedure, but not entirely put into practice.
- ○ The process is defined in practice, but not with a formal procedure
- ○ I do not know

* Who is responsible for the **coordination** of the transition process in your center?

- ○ pediatric rheumatologist
- ○ adult rheumatologist
- ○ adolescent rheumatologist
- ○ internal medicine specialist
- ○ genetician
- ○ other healthcare professional (e.g. nurse, physiotherapist, etc)
- ○ I do not know

* Do you follow any disease-specific **transition guidelines**?

- ☐ No
- ☐ I am not aware about such guidelines
- ☐ Yes

* Who initiates the **transition** process in your center? ❓

- ○ pediatric rheumatologist
- ○ adult rheumatologist
- ○ adolescent rheumatologist
- ○ internal medicine specialist
- ○ genetician

- ○ Other healthcare professional (e.g. nurse, physiotherapist, etc)
- ○ I do not know

* Which is median age of the patient, when you start the **transition** process (talking about transition, encouraging responsibility for the disease,…):

- ○ 10-13 years
- ○ 13-15 years
- ○ 15-18 years
- ○ 18 years old or older.
- ○ I do not know

* How do you assess **transfer readiness**? Select all options you consider relevant

- ☐ Using a validated questionnaire.
- ☐ Personal experience with the patient.
- ☐ Assessment by a psychologist
- ☐ Patient opinion.
- ☐ No assessment, transition process depends on the age of the patient.
- ☐ Others
- ☐ Parents/caregivers opinion
- ☐ I do not know

* Which is the median age of **transfer** to the adult-oriented HCPs?

- ○ Before 16 years.
- ○ 16-18 years.
- ○ 18-20 years old.
- ○ Over 20 years old.
- ○ I do not know

* Which are the factors that influence the **age of transfer**? Select all options you consider relevant

- ☐ End of formal transition program.
- ☐ Patient considered to be ready for transfer by the judgment of the medical team.
- ☐ Low disease activity or remission.
- ☐ Age of patient.
- ☐ End of high school.
- ☐ Medical insurance companies do not cover pediatric clinics anymore.
- ☐ Others
- ☐ I do not know

* Do you have a joint clinic between the pediatric and the adult-oriented team?

- ○ Yes
- ○ No
- ○ I do not know

* Which professionals are involved in the transition process? Select all options you consider relevant

- ☐ Pediatric physician.
- ☐ Adult physician.
- ☐ Pediatric services nurse.
- ☐ Adult services nurse.
- ☐ Social worker.
- ☐ Clinical pharmacist.
- ☐ Geneticists.
- ☐ Physiotherapist or occupational therapists.
- ☐ Oral health professional.
- ☐ Others
- ☐ I do not know

* Is **psychological support** provided during the transition of care?

- ○ Yes,by both the pediatric and the adult-oriented HCPs
- ○ Only by the pediatric HCP
- ○ Only by the adult HCP
- ○ No
- ○ I do not know

* Which topics are **routinely** addressed with the patient during the transition progress? Select all options you consider relevant

- ☐ Understanding of the disease.
- ☐ Genetic basis in heritability.
- ☐ Fertility and parenthood.
- ☐ Sexuality and contraception.
- ☐ Substance use (alcohol/drugs).
- ☐ Medications and treatment compliance.
- ☐ Patients' responsibility for their own health.
- ☐ Mental health and well-being.
- ☐ Work and education.
- ☐ Life expectancy.
- ☐ Preference for adult center.
- ☐ Expectations of adult services.

☐ Others
☐ I do not know

* Do you have a **formal checklist** to discuss these topics during the transition process?

- Yes
- No
- I do not know

* Do you have a **complete** integration of the Electronic health record system between pediatric and adult services (i.e. all visits, radiology, lab results)?

- Yes
- No
- I do not know

* What is included in the t**ransition documentation**? Select all options you consider relevant

☐ Transition letter.
☐ Last visit report.
☐ Recent medications.
☐ Full medical history.
☐ Psychological report.
☐ Education/work plan.
☐ Full medication history.
☐ Sexual health and contraception.
☐ Social habits (smoking, alcohol use).
☐ Specific issues (e.g. needle phobia).
☐ Others
☐ I do not know

* Do you have **dedicated time** to prepare transition documentation?

- Yes
- No

* Do you keep a **database** of transitioned patients?

- Yes
- No
- I do not know

* Do you regularly receive **follow-up information** from adult-oriented HCPs on patients who have transitioned?

- Yes
- No
- I do not know

* From how many different pediatric clinics can your patients be transferred?

- Only 1.
- 2 – 5.
- More than 5.
- I do not know

* Do you have a different practice regarding follow-up according to a juvenile or adult-onset disease?

- Yes
- No
- I do not know

Please specify which specific different practice you make

	Always	Sometimes	Never	Not applicable
Diagnosis name	○	○	○	○
Different assessment of disease activity	○	○	○	○
Different assessment of disease damage	○	○	○	○
Different therapeutical approach	○	○	○	○

The next questions refer to your personal opinion.

How do you rate the efficacy of your transition process?
Reset to initial position

not at all most efficacious

0

‹ ›

0 10

* Which of the following topics are most relevant to address with the patient during the transition process?
Use drag&drop or the up/down buttons to change the order or accept the initial order.

- Genetic basis in heritability.
- Sexuality and contraception.
- Medications and treatment compliance.
- Patients' responsibility for their own health.
- Expectations of adult services.
- Work and education.
- Fertility and parenthood.
- Preference for adult center.
- Life expectancy.
- Substance use (alcohol/drugs).
- Understanding of the disease.
- Mental health and well-being.

* Please rank the weaknesses of your transition program (The most significant weakness should be placed at the top)
Use drag&drop or the up/down buttons to change the order or accept the initial order.

- Abscence of integrated computer system making the documentation preparation too time-consuming
- absence of validated tools to assess outcomes (e.g disease activity, damage..) of pediatric onset connective tissue diseases in adulthood
- Different funding for pediatric and adult care influences medications or treatments after transition
- lack of adolescent training
- lack of guidelines specifically addressing pediatric onset connective tissue diseases diseases in adulthood
- Lack of transition guidelines
- No joint clinics/too little joint clinics
- No suitable specialist adult-oriented HCPs
- Not enough engagement from adult-oriented HCPs
- Not enough/no designated time to prepare documentation
- Pediatric physicians were not motivated in the transition program
- Specific medication or treatments are not available in the adult center
- The caregiver was not ready for the transfer
- The patient was not motivated to transition
- The patient was not ready for the transfer

* Please select from the list the mandatory information that should be provided for an optimum information flow from pediatrics to adult physicians. Select all options you consider relevant

- ☐ Transition letter.
- ☐ Last visit report.
- ☐ Recent medications.
- ☐ Full medical history.
- ☐ Psychological report.
- ☐ Education/work plan.
- ☐ Full medication history .
- ☐ Sexual health and contraception.
- ☐ Social habits (smoking, alcohol use)
- ☐ Specific issues (e.g. needle phobia).
- ☐ Others

* In your opinion, which is the preferred age for starting a **transition process**?

* In your opinion, which is the preferred age for **transfer** to adult-oriented HCPs?

Please describe with 3 words that first come to mind when you think on “transition”

Please give us some of your thoughts on transition care, good practice, pitfalls and possible improvements from y**our personal**

Submit

EUSurvey (/eusurvey/dashboard)

Intellectual Property: *Built by DG DIGIT and funded under the ISA, ISA² (https://ec.europa.eu/isa2/home_en/) and Digital Europe Programme (https://digital-strategy.ec.europa.eu/en/activities/digital-programme) (DIGITAL) EUSurvey is fully open source and published under the EUPL (https://joinup.ec.europa.eu/collection/eupl) licence. You can download the source code from GitHub: https://github.com/EUSurvey (https://github.com/EUSurvey)*

EUSurvey Privacy Statement (/eusurvey/home/privacystatement)
Terms of Service (/eusurvey/home/tos)
Cookies (https://ec.europa.eu/info/cookies_en)

Languages policy (https://commission.europa.eu/languages-our-websites_en)
Privacy policy (https://commission.europa.eu/privacy-policy-websites-managed-european-commission_en)
Legal notice (https://commission.europa.eu/legal-notice_en)

Version v1.5.3.2 bc66c209c78fdbc5b00c1813c8afebcc472dd037 (10/09/2024 09:55)
